# Dynamics of Monolayer Growth in Vapor–Liquid–Solid GaAs Nanowires Based on Surface Energy Minimization

**DOI:** 10.3390/nano11071681

**Published:** 2021-06-26

**Authors:** Hadi Hijazi, Vladimir G. Dubrovskii

**Affiliations:** Faculty of Physics, St. Petersburg State University, 13B Universitetskaya Emb., 199034 St. Petersburg, Russia; dubrovskii.ioffe@mail.ru

**Keywords:** vapor–liquid–solid growth, nanowires, surface energy, monolayer step

## Abstract

The vapor–liquid–solid growth of III-V nanowires proceeds via the mononuclear regime, where only one island nucleates in each nanowire monolayer. The expansion of the monolayer is governed by the surface energetics depending on the monolayer size. Here, we study theoretically the role of surface energy in determining the monolayer morphology at a given coverage. The optimal monolayer configuration is obtained by minimizing the surface energy at different coverages for a set of energetic constants relevant for GaAs nanowires. In contrast to what has been assumed so far in the growth modeling of III-V nanowires, we find that the monolayer expansion may not be a continuous process. Rather, some portions of the already formed monolayer may dissolve on one of its sides, with simultaneous growth proceeding on the other side. These results are important for fundamental understanding of vapor–liquid–solid growth at the atomic level and have potential impacts on the statistics within the nanowire ensembles, crystal phase, and doping properties of III-V nanowires.

## 1. Introduction

The study of semiconductor nanowires (NWs), particularly III-V NWs, has attracted increasing attention in recent years [1]. This interest is due to the morphological advantages of such structures over thin films, related to their high surface to volume ratio and their efficient strain relaxation in contact with a mismatched substrate or within NW heterostructures. III-V NWs are good candidates for a wide range of applications in optoelectronic devices such as solar cells, lasers, LEDs, and single photon emitters, and they can be integrated with a silicon electronic platform. Additionally, NWs allow for an almost unlimited range of material combinations in ternary III-V solid alloys or heterostructures, often inaccessible in planar technologies.

Semiconductor NWs are grown mainly by the vapor–liquid–solid (VLS) method with a metal catalyst droplet assisting the NW formation [2]. The VLS growth proceeds via the mononuclear regime, that is, by successive growth cycles of individual monolayers (MLs) originating from a single nucleus [3,4,5]. Nucleation and growth of NW MLs is fundamentally important as it determines the crystal phase of III-V NWs [5,6], composition of ternary III-V NWs [7,8], and influences the NW doping process [9]. According to the classical view [5,6,10,11], each ML growth cycle is composed of three stages: (i) formation of the critical nucleus, (ii) extension of the ML until the full coverage of the planar solid–liquid interface is reached, and (iii) refill of the droplet with group V atoms to recover the initial supersaturation and nucleate the next ML. In situ growth monitoring of III-V NW growth inside a TEM [12,13,14,15,16] and the corresponding modeling [12,13,15,17,18,19] have substantially refined the old picture. It has been found that (i) the growth interface of zincblende (ZB) NWs is truncated and oscillates in synchronization with the ML growth, providing additional material to complete the ML, and (ii) the growth interface of wurtzite (WZ) NWs is planar. In the latter case, the droplet may not contain enough group V atoms to complete the ML, which is why the fast ML expansion stops at a certain size and then proceeds at a much slower rate of refill with zero supersaturation of liquid.

A pioneering step in understanding of the ML propagation over the whole growth cycle was taken in ref. [14], where in-situ bird’s eye observations of a growing WZ GaAs NW in the self-catalyzed VLS process (with a Ga droplet) were supported by modeling of ML shapes restricted by different edge facets. It was shown that the ML growth is driven by a competition of the $\left\{10\bar{1}0\right\}$ and $\left\{11\bar{2}0\right\}$ facets inside the droplet (with the surface energies $\gamma_{LS}^{10\bar{1}0}$ and $\gamma_{LS}^{11\bar{2}0}$ hereinafter), and the side facet at the triple phase line (TPL) (with the effective surface energy $\gamma_{eff}$ hereinafter). The surface energy $\gamma_{eff}$ contains a contribution from the surface of the liquid droplet and depends on its contact angle [5,6,12,14]. Based on the ML size versus coverage extracted from in situ observations, the energetic and kinetics of ML formation with the stopping effect were considered in ref. [19]. Very importantly, these works gave some estimates for the surface energies of different edge facets of a GaAs ML in the WZ orientation. In particular, $\gamma_{eff}$ was estimated at 0.03 ± 0.01 J·m^−2^, the ratio $\gamma_{LS}^{11\bar{2}0}$/$\gamma_{LS}^{10\bar{1}0}$ was obtained at 1.05 ± 0.01, while $\gamma_{LS}^{10\bar{1}0}=0.123\;J\cdot m^{-2}$ was assumed to be equal to the surface energy of the {110} ZB facet given earlier in ref. [11]. Of course, these values may have some margins due to the error bars in the data or possible uncertainties in the interpretation, and they pertain only to Ga-catalyzed GaAs NWs.

Here, we model the dynamics of ML formation in Ga-catalyzed and Au-catalyzed WZ GaAs NWs growing along the 〈0001〉 direction using a generalization of the approach given in ref. [14]. We show the significant impact of small variations in the surface energies on the ML morphology throughout its growth cycle. Our simulations reveal a possible dissociation of a portion of the already formed ML, which is replaced by simultaneous attachment of another portion of solid on the other side of the ML. This effect, seen in the data of ref. [14], has been previously neglected in modeling. The predicted behavior of the ML growth is important from the fundamental viewpoint as well as for its possible implications for the crystal phase, statistics, composition, and doping of III-V NWs.

## 2. Model

**Figure 1:**
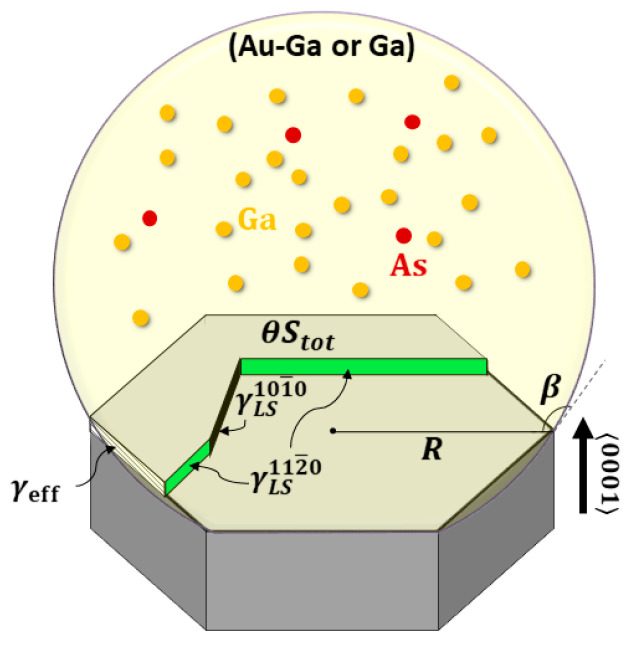
Schematic representation of fractional ML in a WZ GaAs NW, showing the model parameters.

We consider a WZ GaAs NW growing by Ga-catalyzed or Au-catalyzed VLS mechanism along the 〈0001〉 direction. The ML coverage, θ, is defined as the surface area of the growing ML divided by the total surface area of the available solid–liquid interface,$\;S_{tot}=\left(3\sqrt{3}/2\right)R^{2}$, with R as the large radius of the hexagonal NW cross-section. The ML growth starts at θ=0 and ends at θ=1. Here, we consider the surface energy only, which depends on the coverage θ. The volume contribution to the free energy of forming the ML (containing the kinetic factors such as supersaturation) is neglected in the first approximation, as it should be approximately the same for a given θ [9,19]. To calculate the surface energy term of the ML formation energy, $\Delta G_{S}$, we consider nucleation at the TPL, which is necessary for the occurrence of the WZ crystal phase in III-V NWs [6]. In this case, the island (fractional ML) edge at the TPL is formed by adding the vapor–solid (VS) interface with the surface energy $\gamma_{VS}hL_{e}^{10\bar{1}0}$ and eliminating the vapor–liquid (VL) interface with the surface energy $\gamma_{VL}h\sin\beta L_{e}^{10\bar{1}0}$, where $L_{e}^{10\bar{1}0}$ is the length of the external facet at the TPL, β is the contact angle of the droplet, and h is the height of the GaAs ML [5,6,12,14]. The difference between these two quantities equals $\gamma_{eff}hL_{e}^{10\bar{1}0}$. Inside the droplet, two types of edge facets can appear, namely the {$10\bar{1}0$} and {$11\bar{2}0$} facets [14], with six possible orientations for each facet rotated by π/3 with respect to each other, adding the contributions $\gamma_{LS}^{10\bar{1}0}hL_{i}^{10\bar{1}0}$ and $\gamma_{LS}^{11\bar{2}0}hL^{11\bar{2}0}$, respectively. Here, $L_{i}^{10\bar{1}0}$ and $L^{11\bar{2}0}$ are the total lengths of the internal {$10\bar{1}0$} and {$11\bar{2}0$} edge facets. The ML geometry with the three facets of interest is illustrated in Figure 1.

The total change of the surface energy upon forming a fractional ML is given by

(1) $$\Delta G_{S}=\gamma_{eff}hL_{e}^{10\bar{1}0}+\gamma_{LS}^{10\bar{1}0}hL_{i}^{10\bar{1}0}+\gamma_{LS}^{11\bar{2}0}hL^{11\bar{2}0}$$

Our study is based on the minimization of this $\Delta G_{S}$ at any moment of time during the ML growth, related to different coverages θ. This cannot be done analytically due to the non-linearity of Equation (1) as a function of θ. Therefore, for each θ, all possible ML configurations, $\psi_{\theta }$, are calculated numerically, and the configuration that minimizes $\Delta G_{S}$, $\psi_{\theta }^{m}$, is chosen as the energetically preferred shape of the ML at this θ.

The hexagonal top facet of the NW is represented by a regular triangular mesh with spacing between the neighboring nodes l=4 nm. For an NW of radius R=20 nm, this gives sufficient resolution with an increment in θ of ε= 0.007. We limit the number of internal facets to three, meaning that the ML geometry can adopt a polygon shape of *n* facets, with *n* from 3 to 9. Similar to $\psi_{\theta }$, we define $P_{\theta }$ as all possible polygon shapes at the ML coverage θ, with $P_{\theta }^{m}$ as the shape that minimizes $\Delta G_{S}$. The sequence $\Delta_{m}={\left(P_{\theta }^{m}\right)}_{\theta =0}^{1}$ of all the representative polygons at different θ gives the dynamics of ML growth based on the surface energy minimization.

## 3. Results and Discussion

**Figure 2:**
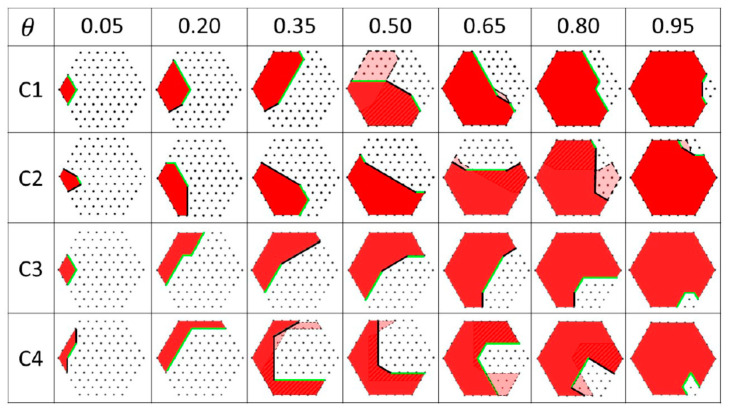
Polygons $P_{\theta }^{m}$ showing the dynamics of ML growth simulated with different sets of surface energies summarized in Table 1. C1 and C2 represent Ga-catalyzed GaAs NW, while C3 and C4 represent Au-catalyzed GaAs NW. The dots represent the nodes of triangular mesh of the NW top facet. This geometry is chosen since it allows us to compute all possible combinations of the inner edge facets of the ML. The {$10\bar{1}0$} and {$11\bar{2}0$} facets are marked by green and black lines, respectively. The opaque polygons represent the extending ML. The transparent polygons show the eliminated ML portions, while dashed polygons correspond to the newly formed ML portions.

The first nucleus of the ML is pinned at one corner of the TPL according to ref. [14]. Its shape is assumed to be a rhombus with side l, which gives the initial coverage $\theta_{0}=l^{2}/\left(3R^{2}\right)=0.0133$. Starting from this value, the coverage is increased by steps as $\theta_{t}=\theta_{0}+t\varepsilon$, with t=1, 2, 3, …, until reaching the full coverage θ=1. For a given $\theta_{t}$, $P_{\theta_{t}}$ are computed and compared, resulting in the energetically preferred $P_{\theta_{t}}^{m}$ extracted for each iteration. These results are shown in Figure 2 for different sets of surface energies given in Table 1. C1 and C2 represent Ga-catalyzed GaAs NWs, while C3 and C4 represent Au-catalyzed GaAs NWs. In Figure 2, we select seven images for each configuration, which correspond to θ= 0.05, 0.2, 0.35, 0.50, 0.65, 0.80, and 0.95.

Let us now discuss the choice of surface energies in Table 1 and the corresponding ML configurations shown in Figure 2. The first set C1 is the same as in refs. [14,19] for self-catalyzed GaAs NWs. The opaque red polygons in the first row of images in Figure 2 represent the deposited ML. The inner {$10\bar{1}0$} and {$11\bar{2}0$} facets are marked by green and black lines, respectively. Our simulation reproduces quite well the experimental observations and modeling of ref. [14]. In particular, the growing ML maintains its rhombus shape restricted solely by the {$10\bar{1}0$} facets until θ = 0.05. After that, it transforms into an irregular polygon with five edge facets (for θ between 0.05 and 0.20), keeping the maximum contact with vapor at the TPL. The {$11\bar{2}0$} facets start to appear only for θ ≥ 0.2. Importantly, we observe the dissociation of a portion of the already formed solid (shown by the transparent red polygons in Figure 2) at θ = 0.5, with the simultaneous formation of a differently shaped portion of solid (dashed zones in Figure 2) on the opposite side of the ML. This effect is repeated several times before the ML completion, as seen, for example, in the C1 image of Figure 2 at θ = 0.65. This phenomenon can be seen in the video SV2 of ref. [14] at different θ, but is not discussed in the paper.

Similar findings have been observed experimentally for ZB NW, where some part of the solid NW is dissolved at the TPL to form truncated facets (whose size is much larger than that of the ML) inside the droplet [12,13,15]. The theoretical explanation for this effect was also based on surface energy minimization, where forming the truncation was energetically preferred within a certain range of droplet contact angles [12]. On the other hand, the truncation provides an additional source of material to rapidly complete the ML. This changes drastically the ML growth dynamics in ZB NWs compared with WZ NWs, where the ML progression slows down at the stopping size due to depletion of As in the catalyst droplet [14,15,19]. We observe the ML reconstruction in WZ NWs, which has not been noticed so far to our knowledge. The fact that our energetic simulations repeatedly predict the rearrangement of material in the growing WZ ML for different sets of surface energies, and correlate with experimental observations, suggests that this effect should be included in the follow-up studies of the NW growth kinetics, particularly at high θ. The ML reconstruction is also possible from the kinetic viewpoint, because dissociation of an ML portion increases supersaturation of liquid in the catalyst droplet (even if it was zero, as occurs when the ML is at its stopping size [19]), which provides the necessary material to rapidly add another portion of solid at a different position.

For comparison, the set of surface energies C2 for Ga-catalyzed GaAs NWs is also considered, which has the same $\gamma_{eff}$ and $\gamma_{LS}^{10\bar{1}0}$ as in C1 but a smaller $\gamma_{LS}^{11\bar{2}0}$. As expected, the {$11\bar{2}0$} facets start to appear earlier due to their lower surface energy compared with C1. Again, reconstruction of an ML portion can be seen at θ = 0.65, and it is repeated at θ= 0.8 and 0.95. The concave shape of the internal ML edges changes to convex for θ≥ 0.35, in contrast to C1 where the concavity is maintained until θ= 0.8. A better correlation with in situ data of ref. [14] is obtained with C1, meaning that $\gamma_{LS}^{11\bar{2}0}$= 0.13 $J\cdot m^{-2}$ gives a better estimate for the liquid–solid surface energy of the {$11\bar{2}0$} facet compared with 0.1$\;J\cdot m^{-2}$.

We now turn to simulations of the ML step flow in Au-catalyzed GaAs NWs. In this case, the values of $\gamma_{eff}$ may be higher, because the liquid–vapor (droplet) surface energy $\gamma_{VL}$ may be increased due to the presence of Au in the droplet [20]. The opposite view is that the droplet surface energy is determined by liquid Ga even for an Au-Ga alloy because Ga should accumulate at the liquid–vapor interface as a lower surface energy metal [15,17], but here we consider a higher $\;\gamma_{eff}$ in the Au-catalyzed VLS process as a possibility. Taking linear interpolation between pure Au and pure Ga droplets, we can calculate $\gamma_{LV}$ as $\gamma_{LV}\approx c_{Ga}\gamma_{Ga}+c_{Au}\gamma_{Au}$. Here, $c_{Ga}$ is the Ga concentration in the Au-Ga alloy, $c_{Au}≅1-c_{Ga}$ is the Au concentration when neglecting As, $\gamma_{Ga}=0.72\;J\cdot m^{-2}$ and $\gamma_{Au}=1.14\;J\cdot m^{-2}$ are the surface energies of pure liquid Ga and Au, respectively [21]. We use two plausible values of $\gamma_{eff}=0.1$ and $0.2\;J\cdot m^{-2}$, corresponding to $c_{Au}=0.25\;$ and 0.5, respectively. We assume that $\gamma_{LS}^{11\bar{2}0}$ and $\gamma_{LS}^{10\bar{1}0}$ keep the same values as in self-catalyzed NWs. These parameters result in the sets C3 and C4 in Table 1. From the ML shapes for C3 and C4 shown in Figure 2, a tendency to develop more internal facets is clearly seen. This result is anticipated at a higher $\gamma_{eff}$ compared with the self-catalyzed VLS process. The ML reconstruction is also present for these configurations.

**Figure 3:**
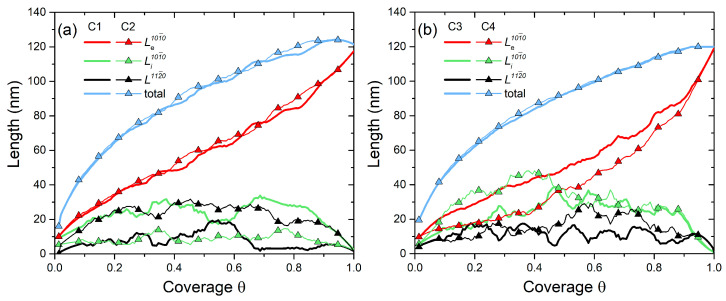
Evolution of lengths of different facets with coverage θ for configurations (**a**) C1 and C2, and (**b**) C3 and C4. In (**a**), $L_{e}^{10\bar{1}0}$ increases quasi-linearly with θ due to a low $\gamma_{eff}$, while in (**b**) its increase is slower. The black and green curves show the interplay between the {$10\bar{1}0$} and {$11\bar{2}0$} facets throughout the ML extension.

To see more clearly the competition between different facets, the evolution of their lengths with θ extracted from the above calculations is shown in Figure 3a for C1 and C2 and in Figure 3b for C3 and C4. For C1 and C2, the low $\gamma_{eff}$ yields a quasi-linear increase in $L_{e}^{10\bar{1}0}$ with θ. These two configurations show very similar evolution of the $L_{i}^{10\bar{1}0}$ and $L^{11\bar{2}0}$. The length of the facet having a lower surface energy prevails all along the ML growth cycle. The same trends are observed for C3 and C4, except for a more nonlinear evolution of $L_{e}^{10\bar{1}0}$ with θ due to a higher $\gamma_{eff}$.

## 4. Conclusions

In conclusion, we have studied the dynamics of ML propagation in WZ GaAs NWs grown by a Ga-catalyzed and Au-catalyzed VLS process. Different sets of surface/interface energies have been tested. It has been shown that a large portion of the already formed GaAs ML can dissolve in liquid during growth, with simultaneous adding of another portion of the ML in a different position. Our study suggests that such reconstruction of the growing ML is mainly governed by the surface energy constraints depending on the ML coverage. We believe that this behavior has an implication on the NW doping properties. This effect will be considered elsewhere.

## Figures and Tables

**Table 1:** Surface energies used for simulations of ML growth.

		$\gamma_{eff}$ $\;(J\cdot m^{-2})$	$\gamma_{LS}^{10\bar{1}0}$ $\;(J\cdot m^{-2})$	$\gamma_{LS}^{11\bar{2}0}$ $\;(J\cdot m^{-2})$
Self-Catalyzed	C1	0.03	0.123	0.13
	C2	0.03	0.123	0.10
Au-Catalyzed	C3	0.1	0.123	0.13
	C4	0.2	0.123	0.13

## Data Availability

Not applicable.
